# An inhibitor of fibroblast growth factor receptor-1 (FGFR1) promotes late-stage terminal differentiation from NGN3+ pancreatic endocrine progenitors

**DOI:** 10.1038/srep35908

**Published:** 2016-10-27

**Authors:** Yzumi Yamashita-Sugahara, Masahito Matsumoto, Manami Ohtaka, Ken Nishimura, Mahito Nakanishi, Kohnosuke Mitani, Yasushi Okazaki

**Affiliations:** 1 Division of Functional Genomics and Systems Medicine, Research Center for Genomic Medicine, Saitama Medical University, Saitama, Japan; 2 Biotechnology Research Institute for Drug Discovery, National Institute of Advanced Industrial Science and Technology (AIST), Ibaraki, Japan; 3 Laboratory of Gene Regulation, Faculty of Medicine, University of Tsukuba, Ibaraki, Japan; 4 Division of Gene Therapy, Research Center for Genomic Medicine, Saitama Medical University, Saitama, Japan

## Abstract

Human induced pluripotent stem cells (hiPSCs) provide a potential resource for regenerative medicine. To identify the signalling pathway(s) contributing to the development of functional β cells, we established a tracing model consisting of dual knock-in hiPSCs (*INS*-Venus/*NGN3*-mCherry) (hIveNry) expressing the fluorescent proteins Venus and mCherry under the control of intrinsic insulin (*INS*) and neurogenin 3 (*NGN3*) promoters, respectively. hIveNry iPSCs differentiated into NGN3- and mCherry-positive endocrine progenitors and then into Venus-positive β cells expressing *INS*, *PDX1*, *NKX6.1*, and glucokinase (*GCK*). Using these cells, we conducted high-throughput screening of chemicals and identified a specific kinase inhibitor of fibroblast growth factor receptor 1 (FGFR1) that acted in a stage-dependent manner to promote the terminal differentiation of pancreatic endocrine cells, including β cells, from the intermediate stage of pancreatic endocrine progenitors while blocking the early development of pancreatic progenitors. This FGFR1 inhibitor augmented the expression of functional β cell markers (*SLC30A8* and *ABCC8*) and improved glucose-stimulated INS secretion. Our findings indicate that the hIveNry model could provide further insights into the mechanisms of hiPS-derived β cell differentiation controlled by FGFR1-mediated regulatory pathways in a temporal-dependent fashion.

Type 1 diabetes is an autoimmune disease characterized by the complete loss of insulin (INS) due to the destruction of β cells, and its treatment is solely dependent on INS administration^1^. Although islet transplantation is a promising therapy for type 1 diabetes, difficulties in obtaining sufficient islets and immunosuppression problems point to the need for alternative cell sources for the generation of INS-expressing β cells^2^. Human induced pluripotent stem cells (hiPSCs) represent an important resource since they have the potential to differentiate into INS-producing functional β cells.

Fibroblast growth factor receptor 1 (FGFR1) signalling during embryonic development is crucial and the phenotype is lethal in *null* mice before or during gastrulation^3^,^4^. Several studies of mouse models have demonstrated the importance of FGFR1 for normal structural development, with FGFR1 mutations linked to Pfeiffer syndrome, which is characterized by abnormal cranial formation^5^,^6^. In pancreatic development, an FGFR1 agonist of FGF4 signalling regulates the patterning of pancreatic and duodenal homeobox 1 (PDX1)-expressing foregut endoderm and pancreatic expansion^7^. Mice expressing dominant-negative FGFR1c driven by the PDX1 promoter develop age-specific diabetes^8^. Moreover, endogenous FGFR1 expression is regulated by PDX1 in β cells, while the expression of a dominant-negative PDX1 mutant inhibits FGFR1 expression leading to the downregulation of Glut2^8^,^9^. Thus, positive feedback regulation via the FGFR1-PDX1 cascade causes the differentiation and maintenance of β cells.

Many studies have reported the generation of pancreatic endocrine cells *in vitro* from human embryonic stem cells (hESCs)/hiPSCs. However, pancreatic β-like cells derived from the differentiation of stem cells *in vitro* exhibit a limited capacity for glucose-stimulated insulin secretion (GSIS), a hallmark of functionally mature β cells^10^,^11^,^12^,^13^. Recently, attempts at generating functional β cells from hESCs/hiPSCs *in vitro* produced PDX1-expressing (PDX1+) pancreatic progenitors (PPs) from definitive endoderm (DE) and maintained functional β cells that featured similar expression profiles and glucose responsivity to primary human β cells under the control of FGFR1-mediated signalling^14^,^15^. According to the differentiation protocols used in these studies, FGFR1 or FGFR2 agonists are utilized to drive the PDX1 expression that is essential for the early stages of β cell differentiation *in vitro*. Therefore, these procedures have been developed mostly based on mimicking normal pancreatic development mediated by the transition from PDX1+ PPs to endocrine progenitors (EPs) transiently expressing neurogenin 3 (NGN3) as a master regulator of hormonal endocrine cells. V-Maf avian musculoaponeurotic fibrosarcoma oncogene homolog A (MAFA), which is expressed together with PDX1 in adult β cells, is critical during the maturation of functional β cells in adult islets. Although the mode of action of FGFs at the early transition from DE to the PDX1+ PP stage is relatively well defined by genetic ablation studies, the regulatory role of FGFR-mediated signalling in the terminal differentiation and maturation processes of hiPSCs -derived β cells and their differentiation instability remains unclear.

To clarify the unresolved issues outlined above, we established a dual fluorescence reporter system using mCherry and Venus to trace the transition of hiPS-derived NGN3+ EP cells to terminally differentiated β cells. The tracing model was designed to facilitate a better understanding of the developmental signals that contribute to the intermediate and terminal differentiation of EPs and lead to the maturation of β cells. Using this reporter system, we have discovered that inhibition of FGFR1-mediated signalling not only enhances the efficiency of endocrine cell intermediate and terminal differentiation, but also improves GSIS while additionally increasing the expression of functional β cell markers. Herein, we propose that blockade of the FGFR1-mediated signalling cascade contributes to the efficient intermediate and terminal differentiation of β cells and the subsequent early PDX1+ PP stage that is essential for the temporal-dependent action of FGFs and the production of functional β cells responsible for the maintenance of glucose homeostasis.

## Results

### Gene targeting at the *INS* and *NGN3* loci in hiPSCs

It has been reported previously that hESCs/hiPSCs have a propensity to differentiate towards certain lineages^16^,^17^ and we tested the ability of the hiPSCs to differentiate into pancreatic endoderm lineages (Fig. S1)^10^. We examined the differentiation efficiency of 246H1 and TIG3/KOSM #7 (TIG) hiPSC lines reprogrammed with OCT4/SOX2/KLF4/Myc via retrovirus and Sendai virus, respectively. Following treatment with activin A and Wnt3a, the mRNA expression of markers of DE (*CXCR4* and *CER1)* and mesendoderm (*Brachyury*) were higher in TIG hiPSCs than in 246H1 hiPSCs from days 1 to 5. Pluripotency markers (*OCT3*/*4* and *NANOG)* were also higher in TIG hiPSCs than in 246H1 hiPSCs during this early period, but were not detected on days 9 and 15. Therefore, we chose the TIG hiPSCs for the construction of knock-in (KI) reporter cells since this line appears to be highly sensitive to mediators of pancreatic β cell differentiation.

**Figure 1:**
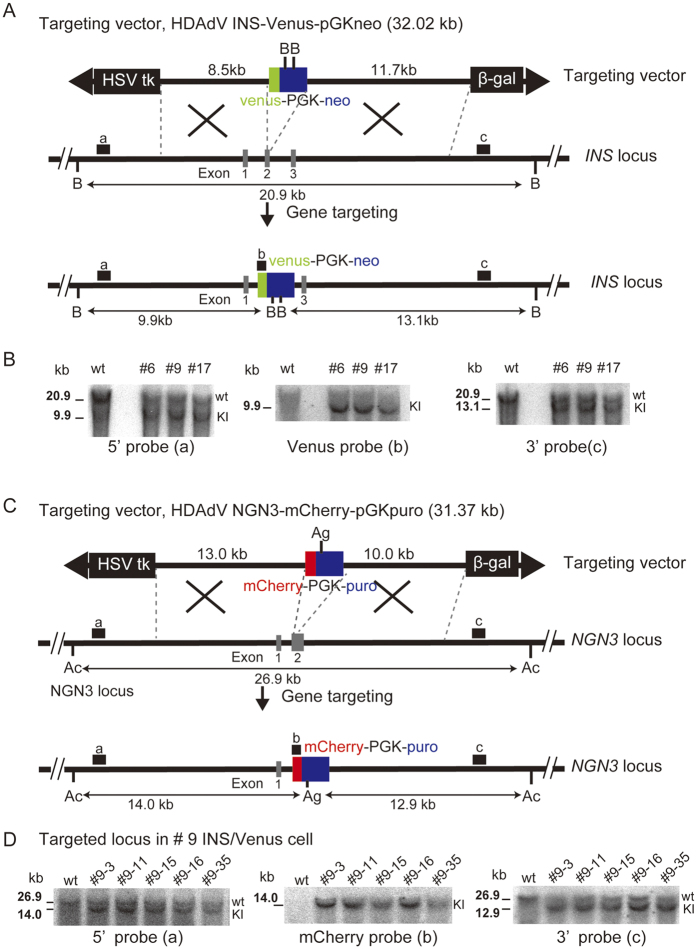
Gene targeting at the INS and NGN3 loci in hiPSCs. (**A**) Schematic illustration of *INS* knockout with HDAdV. The structures of the targeting vector (HDAdV-INS-Ve-pGK-Neo), the wild-type human *INS* locus, and the targeted locus are shown. Venus cDNA was inserted under the *INS* promoter at the ATG of the coding region (at exon 2; exons are shown as grey boxes and numbered 1–3). Venus cDNA: the expression cassette for Venus (yellow fluorescent protein gene). HSV*tk*: the herpes simplex virus thymidine kinase gene cassette. *Bst*Xl sites (B) are indicated; probes a, b, and c indicate the 5′ probe, Venus probe, and 3′ probe used for Southern blotting analysis, respectively. (**B**) The targeted hiPSC clones (#6, 9, and 17) were analysed by Southern blotting with the 5′ probe (a) (9.9 kb), Venus probe (b) (9.9 kb), and 3′ probe (13.1 kb). (**C**) To target NGN3-mCherry, we chose clone #9 of the INS-Venus-targeted hiPSCs. The structure of the targeting vector for NGN3-mCherry (HDAdV-NGN3-mcherry-pGK-puro) is shown. mCherry cDNA was inserted under the control of the *NGN3* promoter at ATG of the coding region (at exon 2; exons are shown as grey boxes numbered 1 and 2). *Acc*I (Ac) and *Age*I (Ag) sites are indicated, and probes a, b, and c indicate the 5t probe, mCherry probe, and 3p probe used for Southern blotting analysis, respectively. (**D**) The targeted hiPSC clones (#9–3, 9–11, 9–15, 9–16, and 9–35) were analysed by Southern blotting analysis with the 5′ probe (a) (14.0 kb), mCherry probe (b) (14.0 kb), and 3′ probe (c) (12.9 kb). Uncropped versions of the scans are presented in Supplementary Fig. S6. HDAdV: helper-dependent adenoviral vector.

We constructed a helper-dependent adenovirus targeting vector (HDAdV) to generate KI hiPSCs that marks INS-producing cells with the green fluorescent protein Venus (*INS*-Venus) (Fig. 1A). TIG hiPSCs were infected with adenoviral particles packed with INS-Venus-PGKneo HDAdV, and drug-resistant clones were analysed by Southern blotting to determine gene-targeted clones (Fig. 1B). The parental TIG (wt) and three selected clones (#6, 9, and 17) are shown with the wild-type *INS* allele displaying a 20.9-kb band on *Bst*XI-digested DNA using a 5i or 3o probe (Fig. 1A,B, probes a and c) and the *INS*-Venus KI locus showing a 9.9-kb band with a 5V probe and a 13.1-kb band with a 3′ probe (Fig. 1B, KI). Among the three KI clones (#6, 9, and 17), one (#9) was chosen to generate the dual KI (DKI) clones expressing the red fluorescent protein mCherry driven by the neurogenin 3 (NGN3) promoter (*NGN3-*mCherry) (Fig. 1C). Southern blot analysis indicated that homologous recombination in the five representative clones (#9–3, 9–11, 9–15, 9–16, and 9–35) occurred properly and efficiently using the NGN3-mCherry HDAdV system, based on the detection of 14.0-kb and 12.9-kb bands following digestion of genomic DNA with *Acc*I (Ac)/*Age*I (Ag) using the 5′, mCherry, and 3 probes (Fig. 1C,D, probes a, b, and c, respectively). The DKI clones we established above are herein termed hiPS/*INS*-Venus/*NGN3*-mCherry (hIveNry) cells.

### Tracing the differentiation of hIveNry DKI cells into pancreatic endocrine lineages

**Figure 2:**
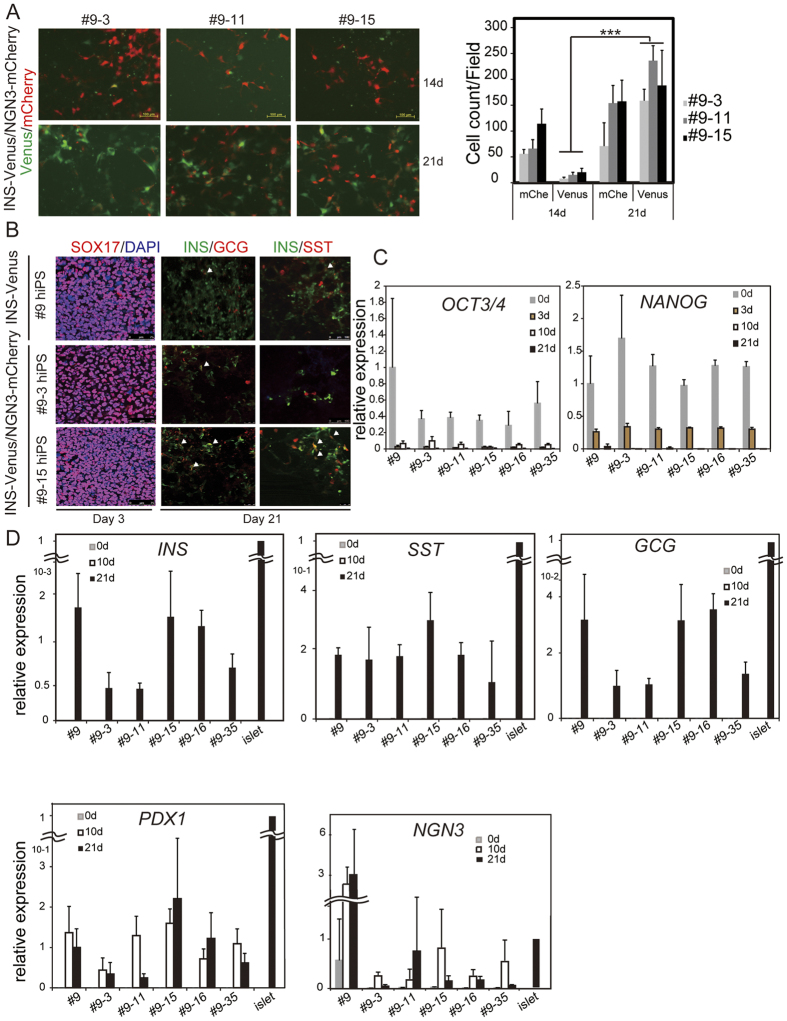
DKI INS-Venus/NGN3-mCherry hiPSC (hIveNry) clones can differentiate into pancreatic endocrine cells. (**A**) hIveNry cells were differentiated towards pancreatic β cells and analysed by *in vivo* imaging on days 14 and 21 of differentiation. The graph shows the reporter-positive cells at the indicated days. (**B**) hIveNry cells were analysed by immunofluorescence on day 3 for the expression of the definitive endoderm marker SOX17 and, on day 21, the final differentiation day, for the co-expression of INS and GCG or INS and SST. mRNA expression analysis of hIveNry clones on days 0, 3, 10, and 21 of differentiation shows the pluripotency markers *OCT3/4* and *NANOG* (**C**) and the endocrine markers *INS*, *SST*, *GCG*, *PDX1*, and *NGN3* (**D**). d: day; SOX17: sex-determining region Y (SRY) box 17. Scale bar, 100 μm.

To test the potential of DKI hiPSC clones to differentiate into pancreatic β cells, we used a previously reported protocol to produce NGN3+ EP and INS-producing cells with high efficiency (Takeda’s protocol, Fig. S2A)^13^. Takeda’s protocol consists of three steps: differentiation of hiPSCs to DE by treatment with CHIR and activin A (days 1–3, stage 1); production of PPs by treatment with dorsomorphin, retinoic acid (RA), and SB431542 (days 3–10, stage 2); and differentiation into endocrine cells by treatment with forskolin (Fsk), dexamethasone (Dex), nicotinamide (Nico), and an Alk5 inhibitor (days 11–21, stage 3). We observed mCherry-positive (mCherry+) cells in each of the three hIveNry DKI clones (#9–3, 9–11, and 9–15) just after the appearance of PPs (early in stage 3, around day 11), and the number of mCherry+ cells was augmented at day 14 as visualized under a microscope (Fig. 2A). Furthermore, a small number of Venus-positive (Venus+) cells could also be detected. mCherry+ and Venus+ cells were counted and, as shown in Fig. 2A (right panel), there were more Venus+ cells on day 21 than on day 14 (Supplementary Video). In Fig. 2B, immunofluorescence analysis shows the parental #9 hiPSCs (INS-Venus KI hiPSCs) and DKI hiPSCs derived from the #9 cells, named #9–3 and #9–15; on day 3 of differentiation, we could detect the expression of SOX17, which is a marker of DE. Moreover, on day 21, we could detect INS, a marker of β cells, glucagon (GCG), a marker of alpha cells, and somatostatin (SST), a marker of δ cells. Some of the double-stained cells co-expressed INS and GCG or INS and SST, indicating immature pancreatic β cells (Fig. 2B).

**Figure 3:**
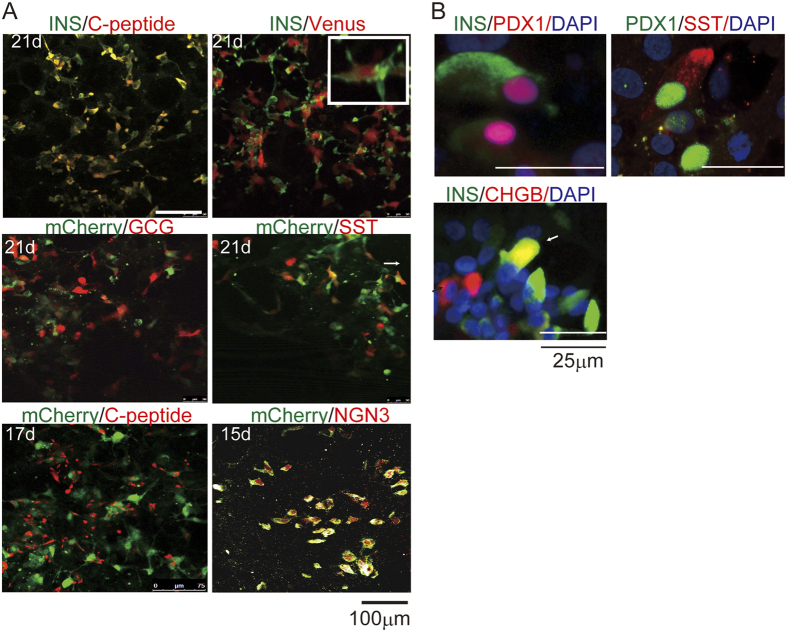
Expression of the fluorescent marker proteins Venus and mCherry coincides with INS and NGN3 expression, respectively. One of the hiPSC clones (#9–15) was selected for immunostaining analysis after differentiation into pancreatic endocrine cells. (**A**) Venus and INS proteins were detected on day 21 with an anti-GFP antibody and anti-INS antibody, respectively. A magnified image of the INS and Venus double-stained cells is shown. mCherry protein was detected with an anti-RFP antibody and co-stained with an anti-NGN3 antibody on day 15. INS expression was analysed with an anti-INS antibody and anti-C-peptide antibody to view the coincidence of INS expression (days 21 and 17). Co-staining of mCherry with GCG, mCherry with SST, and mCherry with C-peptide was analysed. (**B**) On the final day of differentiation (21 days), immunostaining was performed for other markers, namely, PDX1 with INS, PDX1 with SST and INS with CHGB. Scale bar, 100 μm in A and 25 μm for B.

The Venus KI hiPSCs (#9) and their clones displayed similar potential for differentiation. The mRNA levels of the pluripotency markers *OCT3*/*4* and *NANOG* were high in hiPSCs prior to induction at day 0, decreased sharply by day 3, and were undetectable on days 10 and 21 (Fig. 2C). mRNA for the endocrine markers *INS*, *GCG*, and *SST* was detectable on day 21, but not on days 0 or 10, indicating that all DKI hIveNry clones are capable of differentiating into α, β, and δ cells (Fig. 2D) and also into pancreatic polypeptide-expressing and ghrelin-positive ε cells (data not shown). The PP marker *PDX1* was also upregulated on day 10 during the early PP stage and terminal late-stage on day 21. The transient expression pattern of the EP marker *NGN3* in clones #9–15 and #9–35 was also strikingly similar to the normal development of NGN3+ EPs *in vivo* (Fig. 2D). Co-staining of the INS and C-peptide matched fully and the expression of INS and Venus coincided, indicating that the Venus-expressing cells could be monitored as INS-positive (INS+) cells (Fig. 3A). Furthermore, immunostaining analysis revealed that mCherry+ cells coincided with NGN3-expressing EP cells (Fig. 3A). To characterize insulin-producing and other endocrine cells generated by this protocol, we examined the expression of some of the beta cell lineage markers, such as PDX1 and CHGB. As shown in Fig. 3B, PDX1 and CHGB were expressed with INS in most cells, but PDX1 was also expressed with SST in some of the cells. In addition, CHGB was additionally expressed in other types of cells not expressing INS (Fig. 3B). Together, these results indicate that several hIveNry DKI clones (#9–3, 9–11, 9–15, 9–16, and 9–35) could be used to trace the terminal differentiation of the β cell lineage from mCherry+ EP cells (EPs).

### Analysis of Venus+ and mCherry+ EP cells

**Figure 4:**
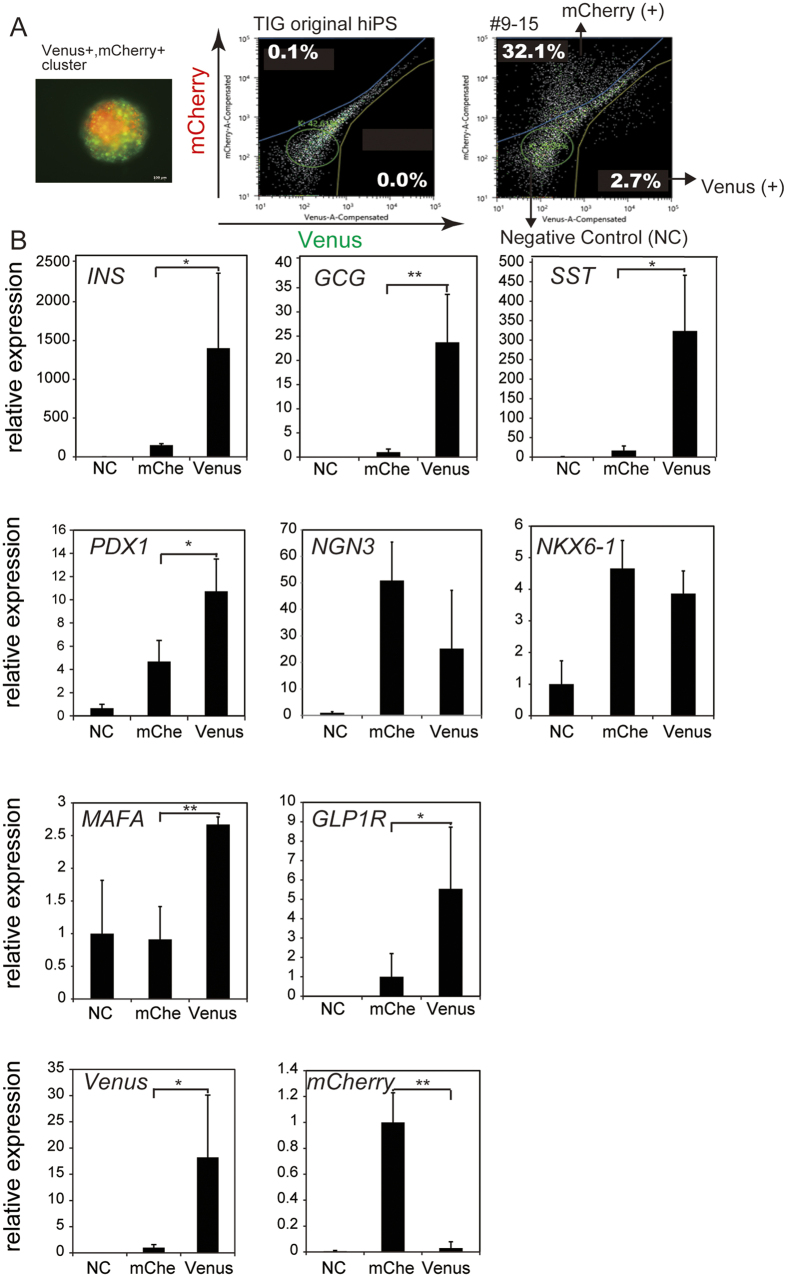
Differentiated DKI hIveNry #9–15 Venus-expressing cells are polyhormonal β cells. DKI hIveNry (clone #9–15) hiPSCs were differentiated into pancreatic β cells. Venus+ cells, mCherry+ cells (mche), and negative control (NC) cells were sorted with a cell sorter (SH800Z), and the mRNA expression levels of each fraction of cells were analysed to characterize the differentiated cells. (**A**) The live cell picture shows differentiated β cells before sorting at 21 days of differentiation, with Venus+ and mCherry+ and double-positive cells evident in the clusters. Gating of Venus and mCherry for sorting. The left picture indicates the negative cells that do not express the reporter genes. The right side shows positive cells and the gated population. (**B**) The mRNA expression levels of the sorted samples (*INS*, *GCG*, *SST*, *PDX1*, *NGN3*, *NXK6.1*, *MAFA*, *GLP1R*, Venus, and mCherry) with data from 3 independent experiments presented as the mean ± standard deviation (SD). NKX6.1: Nirenberg and Kim homeobox 6.1; MAFA: V-Maf musculoaponeurotic fibrosarcoma oncogene homolog a; GLP1R: glucagon-like peptide 1 receptor. *p < 0.05; **p < 0.01.

As reported, the efficiency of differentiation into pancreatic β cells from hiPSCs is approximately 10% from INS-producing cells^13^. Therefore, it is desirable to profile each group of fluorescent cells to view their differentiation state. To profile each group of fluorescence-positive cells, differentiated hIveNry cells were sorted on day 21. Venus+ cells, mCherry+ cells, and fraction of cells with no fluorescence (as a negative control) (Fig. 4A) were characterized by analysing the mRNA levels of several endocrine markers (Fig. 4B). Importantly, Venus+ cells were highly enriched for markers of the β cell lineage, such as *INS*, *PDX1*, *GLP1R*, and *MAFA*, in addition to *GCG* and *SST*. By contrast, mCherry+ PE cells were more enriched for *NGN3* than Venus+-sorted cells (Fig. 4B), in accordance with a mutually exclusive relationship between NGN3 and PDX1^18^,^19^ (Fig. S4B). Although Takeda’s protocol is incapable of producing mature β cells from iPSCs and some of the INS+ cells represent immature polyhormonal cells, we found that the sorted Venus+ cells co-expressed *GCG* and *SST* together with *INS*. Also, we observed that both mCherry+- and Venus+-sorted cells expressed *NKX6.1*, a progenitor marker that is also expressed in mature β cells^14^,^15^. These data provide a strong rationale for investigating Venus+ and mCherry+ cells as potential EP and INS-producing cells for their utility in elucidating the mechanisms of β cell differentiation from hiPSCs.

### High-throughput chemical screening

**Figure 5:**
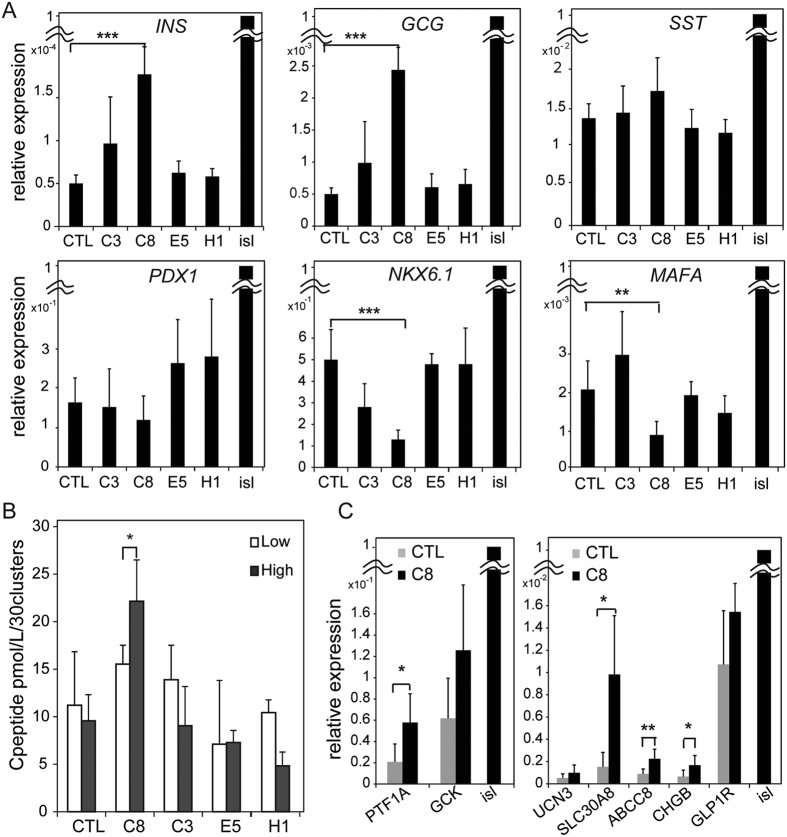
Contribution of C8 to the maturation of functional β cells including their acquisition of glucose sensitivity. (**A**) #9–15 hIveNry cells were plated into V-bottom plates and differentiated in the same manner as was used in the screening assay. The cells were treated with the chemicals and screened between days 10 and 21 of differentiation. On day 21 of differentiation, 29 to 30 clusters from each group were harvested and analysed for the mRNA expression levels of the endocrine markers *INS*, *GCG*, *SST*, *PDX1*, *NKX6.1*, and *MAFA*. (**B**) We tested glucose responsiveness with 30 clusters that were collected and plated into 1 well of a 24-well plate for the GSIS test with sequential methods. The samples were washed 3 times with PBS, pre-incubated in KRBH/2.8 M glucose buffer for 1 h, washed 3 times with PBS, and then incubated in low-concentration glucose (2.8 mM) for 1 h. After the supernatant liquid was sampled and the cells were washed twice with PBS, incubation was continued with high glucose (25 mM). The samples were analysed using an Ultrasensitive C-peptide ELISA kit (Mercodia). (**C**) The expression of the maturation-related markers *UCN3*, *SLC30A8*, *CHGB*, *GLP1R*, *ABCC8*, *PTF1A,* and *GCK* in 30 clusters between the control (DMSO) and C8-treated samples was assessed. Error bars indicate SD, n = 3. *UCN3*: urocortin 3; *SLC30A8*: solute carrier family 30 member 8; *PTF1A*: pancreas transcription factor 1A; *CHGB*: chromogranin B; *ABCC8*: ATP-binding cassette transporter sub-family C member 8; *GCK*: glucokinase; isl (islet). *p < 0.05; **p < 0.01.

The hiPSC-derived INS-producing cells used in this study had no glucose responsivity^13^. In an effort to achieve glucose responsiveness and improve the efficiency of the terminal differentiation of β cells, we screened 90 chemicals commercially available from LOPAC Pfizer (Sigma-Aldrich) (Table S2), as shown in Fig. S2A. In the first quantitative high-end imaging screen using the detection of Venus+ INS-producing cells, we identified 9 chemicals (C3, C7, C8, C10, G11, E5, F8, H1, and H11), 6 of which inhibit tyrosine kinases (C3, C7, C8, C10, E5, and G11) (see Supplementary Table S2). After a second round of screening by quantitative RT-PCR analysis, we selected 3 chemicals (C3, C8, and E5) that seemed to promote β cell differentiation based on the expression of the endocrine markers *INS*, *GCG*, *SST*, *GLUT2*, and *MAFA*, which are critical in establishing β cell function (Fig. S2B,C). We also treated the cells with a specific inhibitor of Notch signalling (H1), a pathway that is indispensable for normal pancreatic development^20^, but this inhibitor did not promote differentiation. We observed quantitative reproducible findings for 4 candidates: C3, C8, E5, and H1. However, we concluded that the compound with the most significant efficacy in differentiating endocrine cells was C8 (PD166866), a specific inhibitor of FGFR1 kinase (Fig. 5A). Importantly, a third screening treatment with C8 alone was sufficient to significantly improve INS secretion in response to high glucose levels (Fig. 5B). This unexpected finding prompted us to examine whether treatment with C8 could affect the expression of functional β cell markers directly linked to INS secretion, as well as other markers related to β cell development and function. Strikingly, several such β cell markers, including *SLC30A8* (zinc transporter), *ABCC8 and* chromogranin B (*CHGB*), were significantly increased by treatment with C8 (Fig. 5C). These results suggest that specific blockade of FGFR1 signalling promotes the intermediate and terminal differentiation of endocrine cells.

### Augmentation of pancreatic endocrine cell differentiation by a specific inhibitor of FGFR1

Previous studies have shown that FGFR1-mediated signalling is essential for the differentiation of DE to PPs towards β cell maturation^12^,^21^. Indeed, most protocols for making β cells from stem cells utilize the FGFR1 agonists bFGF and FGF4 together with stimulation by RA to drive PDX1 expression towards the PP stage. Therefore, our observation that inhibition of FGFR1 signalling by C8 significantly augmented β cell lineage marker expression is unexpected and appears to be paradoxical. However, one possibility is that FGFR1 signalling promotes the early PP stage but inhibits the intermediate stage of β cell differentiation. To test this, we examined the effects of an FGFR1 inhibitor on the early PP stage or intermediate stage of differentiation. We treated the cells with C8 during the early stages, from days 3 (DE) to 10 (PPs), or during intermediate and terminal differentiation, from days 10 (EPs) to 21. Intriguingly, the addition of C8 during the early stages completely inhibited the expression of a limited set of endocrine markers—*INS*, *GCG*, *SST*, *PDX1*, and *NKX6.1,* (Fig. S3A,B, [ii]). To determine if the FGFR1 signalling was responsible for the effect of C8, we evaluated the effects of *shFGFR1* treatment at an early stage. As expected, *shFGFR1* treatment downregulated *PDX1* expression (Fig. S5C). To our surprise, the addition of the inhibitor at the intermediate-terminal stage had the opposite effect and induced a significant increase in endocrine markers (Fig. S3B, [i]). Thus, the appearance of a significant difference in the mode of FGFR1-mediated action seems to be dependent on the temporal stage of differentiation.

**Figure 6:**
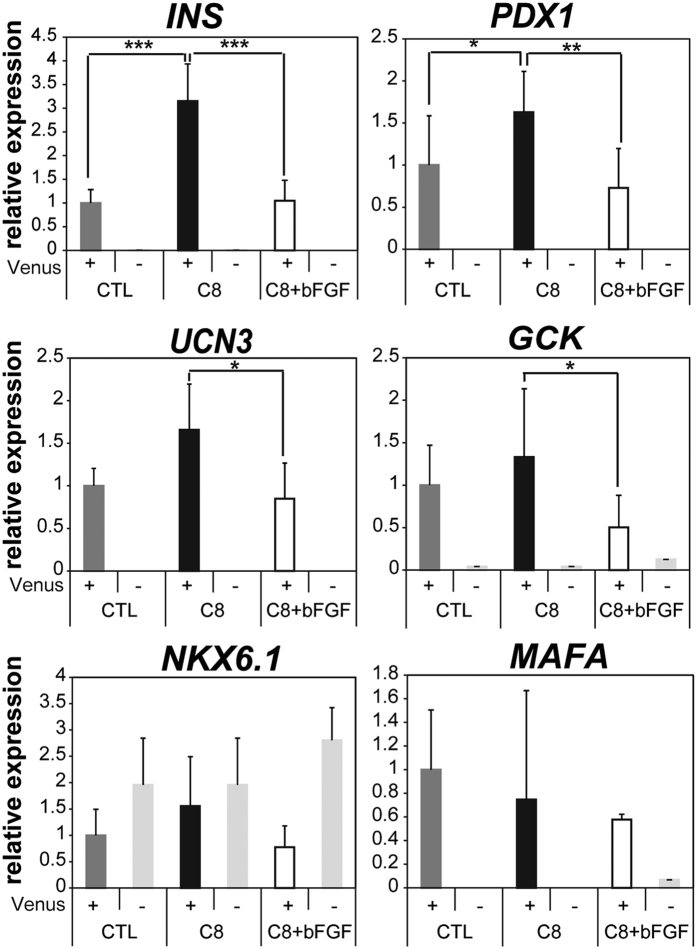
C8 augments important maturation markers specifically in INS-producing β cells. Quantitative RT-PCR analysis was performed to measure the relative expression levels of the β cell lineage markers *INS*, *UCN3*, and *GCK* and the transcription factors *PDX1*, *NKX6.1*, and *MAFA* from sorted Venus+ and NC cells with or without treatment with C8 and/or bFGF. Briefly, #9–15 hiPSCs were differentiated as described above and treated with 5 μM C8 or 5 μM C8 and 50 ng/mL bFGF in the third stage of differentiation. After differentiation, samples were dissociated with TrypLE and sorted with an SH800Z cell sorter (Sony) for Venus+ and NC cells. Fifty cells from each group were sorted into 5 μL FCP reagent, which is a lysate reagent of the QIAGEN Fast Lane cDNA kit. For cDNA synthesis, 2 μL of the samples were used, with 0.5 μL of the 10 μL cDNA samples used for quantitative RT-PCR. Error bars indicate SD, n = 3. *p < 0.05; **p < 0.01; ***p < 0.001.

To better understand the effects of a blockade of FGFR1-mediated action, we verified the efficiency of β cell differentiation from EPs by C8 treatment using the hIveNry system (Fig. S4). Fluorescence-activated cell sorting analysis showed that treatment with the inhibitor substantially increased the size of both the mCherry+ EP cell population and the Venus+ β cell population by 2.3-fold and 3.4-fold, respectively. This increase in cell population size was significantly attenuated by treatment with the FGFR1 agonist bFGF (Fig. S4B). Similarly, the expression of *INS* and *PDX1* genes was substantially higher in sorted Venus+ cells treated with C8 than in untreated control cells (Fig. 6). Furthermore, augmentation of a series of β cell lineage markers by C8, including *INS*, *PDX1*, urocortin 3 (*UCN3*), and *GCK*, was specifically attenuated by the addition of the FGFR1 agonist bFGF (Fig. 6, C8 + bFGF). The expression levels of *NKX6.1* and *MAFA* were not affected by treatment with C8 and bFGF. FGF regulation of the intermediate-stage differentiation of β cells appears to be mediated selectively by FGFR1 since treatment with the FGFR2-specific agonist KGF did not affect the ability of C8 to increase the levels of β cell lineage markers (data not shown). All of our results are compatible with previous reports indicating that FGFR1-mediated signalling is necessary for the early differentiation of PPs. However, we made the surprising discovery that blockade of FGFR1 signalling robustly improves pancreatic endocrine cell differentiation at later stages of endocrine intermediate and terminal differentiation, indicating that FGFR1-mediated signalling has opposing effects on cell fate specification that are stage-dependent.

## Discussion

It has been reported recently that *in vitro* differentiation protocols improve the maturation of pancreatic β cells^14^,^15^. Many studies reported that this was due to the presence of maturation factors and compared the differential expression of these factors in β cells from mice or humans that were immature or mature and either foetal or adult^22^,^23^,^24^,^25^. However, the molecular mechanisms of β cell differentiation remain largely unknown and protocols for generating β cells from stem cells *in vitro* have not been completely defined. In this study, we established DKI hiPSCs (hIveNry) that could be used to trace the differentiation status of pancreatic β cells. Using these cells, we discovered that inhibition of FGFR1 signalling not only promotes the intermediate stage of endocrine cell differentiation from EPs, but also contributes to their terminal differentiation towards mature functional β cells.

FGF signalling is indispensable for normal pancreatic development^26^,^27^,^28^. Although many studies have demonstrated the importance of FGFR1 signalling during development, the phenotypes of mice lacking FGFR1 or its agonist FGF4 do not address the role of FGFR1 in endoderm formation and patterning^29^. Our hIveNry tracing model allowed us to gain molecular insights into how FGFR1-mediated signalling plays roles in each stage of the early- and/or intermediate-stage differentiation of β cells. We confirmed the findings of previous studies by demonstrating that treatment with the FGFR1 inhibitor C8 abrogates the early developing PP stage and the expression of *PDX1* (Fig. S3). Several lines of evidence support a role for an FGFR1-PDX1 feedback loop in the development of PPs from DE. First, the FGFR1 agonist FGF4 induces the expression of PDX1 as a target gene^27^. Second, mice expressing a dominant-negative FGFR1 driven by the *PDX1* promoter become diabetic due to the loss of β cells and the expression of *Glut2*^8^. Third, overexpression of dominant-negative PDX1 attenuates FGFR1 expression *in vivo*^9^. Finally, ESCs lacking FGFR1 are unable to develop into pancreatic lineages^30^. These findings suggest that positive feedback regulation of the FGFR1-PDX1 cascade is essential for early β cell development *in vitro* and *in vivo*. In fact, the expression of *PDX1* and *FGFR1* is concordantly higher at the PP stage, and their expression profiles are correlated at each distinct stage (Fig. S5A). Moreover, this hypothesis was strongly supported by knockdown experiments showing that *shPDX1* suppresses *FGFR1* expression and that *shFGFR1* suppresses *PDX1* expression (Fig. S5C).

In contrast, the expression of *FGFR2* appears to be lower than that of *FGFR1*. Therefore, it is reasonable to conclude that the expression of *PDX1* is tied to that of *FGFR1* since *PDX1* is a downstream target of *FGFR1*. Thus, the FGFR1-PDX1 cascade is required to drive the transition towards the early PP and DE stages during pancreatic development and to establish β cell function. However, we cannot rule out the possibility that alternative signals are necessary for sustaining PDX1 expression in adult β cells.

**Figure 7:**
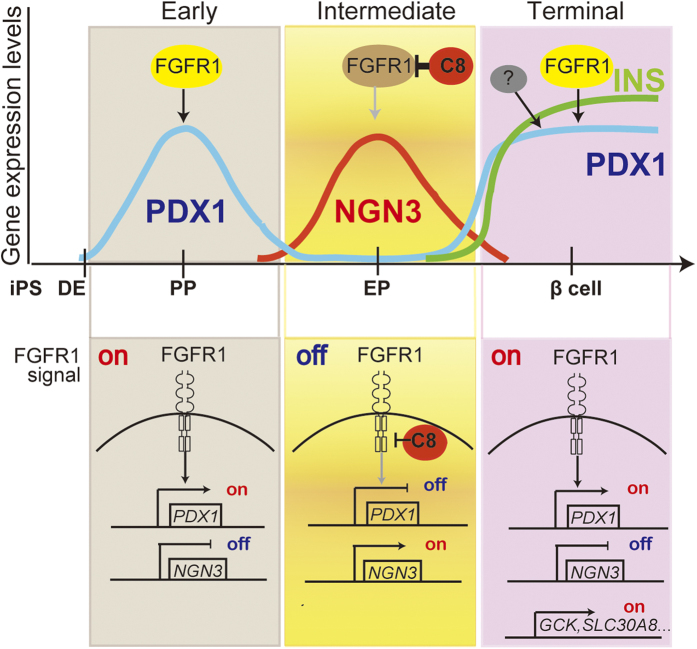
Modelling of the proposed role of FGFR1 signalling during the early and intermediate stages of pancreatic β cell differentiation. Pancreatic β cells are derived from hiPSCs/ESCs via multiple differentiation stages including definitive endoderm (DE), pancreatic progenitor (PP), and endocrine progenitor (EP). On the basis of the characteristics of the expression profiles of PDX1 and NGN3 as master regulators of whole pancreas and hormonal EPs, respectively, β cell differentiation can be divided into at least 3 stages. First, the early stage is initially responsible for cell fate specification of the pancreas by the expression of PDX1 in PPs stimulated by FGFR1-mediated actions from DE. Second, the intermediate stage of differentiation can be classified as the transition from the downregulation of PDX1, prior to giving rise to NGN3-expressing EPs, to INS-producing cells after the loss of NGN3 expression. Third, the terminal process of β cells establishes β cell functions including glucose-stimulated insulin secretion (GSIS) and maintenance of glucose homeostasis by sustaining the expression of PDX1 in mature β cells, possibly due to the activation of FGFR1-mediated and/or other signals. We hypothesize that inhibition of FGFR1 signalling by C8 might promote efficient intermediate-stage differentiation of NGN3+ EPs towards functional mature β cells. The upper panel shows the temporal relationships between the processes of β differentiation along with INS expression and the expression levels of the transcription factors NGN3 and PDX1 as downstream targets of FGFR1-mediated signalling. The lower panel provides a schematic representation in which the mode of intracellular FGFR1 signalling and its distinct status at each differentiation stage are shown. According to this representation, the mutually exclusive relationship between the expression of NGN3 and PDX1 depends on differentiation status and FGFR1 signalling or its inhibition by C8.

Prior to this study, the role of FGFR1-mediated signalling in the intermediate NGN3+ EPs stage towards β cell differentiation was unknown. We showed that while FGFR1-mediated signalling is indispensable for the development of early-stage PPs from DE, blockade of FGFR1 at the intermediate stage of β cell differentiation is critical for the acceleration of efficient endocrine cell differentiation (Figs 5, 6, S3, and S4). Surprisingly to us, the selective augmentation of β cell differentiation by the inhibition of FGFR1-mediated signalling seems to play a novel and distinct role in the development of EPs. Our results are unexpected since they appear to contradict evidence showing that FGFs are crucial for β cell development, as described above. Rather, the results presented here are consistent with previous studies and reveal distinct and opposing roles for FGFR1 signalling during early PP differentiation as opposed to intermediate-stage differentiation. In fact, in sorted mCherry+ EPs, the expression of PDX1 was diminished relative to that observed in PPs (Figs 4B, and S5A), whereas NGN3 was highly expressed in mCherry+ cells (Fig. S5B). This suggests that PDX1 is no longer necessary for the intermediate-stage differentiation of NGN3+ EPs based on evidence of the mutually exclusive relationship between PDX1 and NGN3. This may partly explain why blockade of the FGFR1-PDX1 cascade contributes to the efficient β cell differentiation of NGN3+ EP cells. Therefore, based on our results, we hypothesize a temporal switching model in which FGFR1 signalling drives PDX1 expression in the early stage and in mature β cells (as shown in Fig. 7). To our knowledge, this is the first demonstration that inhibition of FGFR1 signalling drives the augmentation of both NGN3+ EP cell and β cell differentiation. Indeed, this idea is clearly supported by the potentiation of Venus+ β cell (3.4-fold) and mCherry+ progenitor cell (2.3-fold) differentiation upon treatment with C8 and by the ablation of these effects in the presence of a supramaximal dose of the FGFR1 agonist bFGF (Fig. S4B). Further investigation will clarify the molecular mechanisms underlying the inhibitory effect of FGFR1 signalling during intermediate-stage differentiation towards β cell function.

Although the FGFR1 agonists bFGF, FGF2, and FGF4 are necessary for early PP development to induce PDX1 expression, FGFR1 signalling might be attenuated to facilitate transition to the NGN3+ EP stage, which is mutually exclusive with the expression of PDX1 during intermediate-stage differentiation. Regarding FGFR2-mediated signalling, a mouse model lacking FGF10 showed abrogation of pancreatic organogenesis and impairment of the maintenance and expansion of PDX1+ cells^26^. These results are intriguing and prompted us to determine whether FGFR2 signalling plays a role in the intermediate-stage differentiation of β cells. Apparently, this is not the case since treatment with the FGFR2 agonist KGF was unable to significantly alter the effects of C8 on endocrine differentiation (data not shown). In contrast, bFGF treatment attenuated β cell differentiation and the expression of the associated lineage markers *INS*, *PDX1*, *UCN3*, and *GCK*, strongly indicating that FGFR1 but not FGFR2 signalling regulates the selective and specific cell fate specification of the intermediate-stage differentiation of β cells, possibly to drive PDX1 expression in a temporally determined, stage-specific manner (Figs 6 and S5). The distinct roles of FGFR1 and FGFR2 might be explained by evidence that the FGFR2 agonist FGF10 is essential for the proliferation of PDX1+ cells rather than for the induction of PDX1 as an initial driver^26^.

Tyrosine kinase inhibitors inhibit GSIS capacity^31^ and the majority of compounds identified by our screen target tyrosine kinases (Table S2). Among them, we found that treatment with C8 accelerated GSIS (Fig. 5B), possibly by increasing the levels of functional β cells due to the increased expression of functional markers, including *SLC30A8* and *ABCC8* (Fig. 5C). These results are similar to those demonstrating that the selective tyrosine kinase inhibitor imatinib promotes INS release from β cells^32^. In addition, the blockade of tyrosine kinase signalling, such as Src family kinases and FAK, can promote β cell differentiation^33^. Thus, there is growing evidence that tyrosine kinase signalling has a role in the differentiation of β cells as well as in maintaining the function of mature β cells. However, further study is needed to elucidate the roles of tyrosine kinases in this context and the molecular mechanisms involved.

In conclusion, we have made the unexpected discovery that specific inhibition of FGFR1 signalling at the intermediate stage promotes efficient later-stage (intermediate and terminal) differentiation of endocrine cells.

## Materials and Methods

### Construction of the *INS*-Venus and *NGN3*-mCherry bacterial artificial chromosome (BAC) clones and the pHDAdV *INS*-Venus-pGKneo and pHDAdV *NGN3*-mCherry-pGK-puro vectors

The human BAC clones CH17-182P12 and RP11-343J3 contain the human *INS* and *NGN3* gene loci, respectively, from the CH17 and RP11 Human BAC Library (BACPAC Resources, Oakland, CA, USA). The BAC-based targeting vector was generated as previously reported^34^. To construct the human *INS*-Venus and *NGN3*-mCherry KI constructs, the BAC clones were modified by using the RED/ET recombination technique^35^. The Venus-loxP-PGK-EM7-neo-bpA-loxP cassette was inserted into the CH17-182P12 BAC clone and the mCherry-loxP-PGK-EM7-neo-bpA-loxP cassette was inserted into the target site of the RP11-232J3 BAC clone. Subsequently, a total of 22–23 kb of homology, including the marker cassette, was sub-cloned into the HDAdV plasmid. A detailed description of these sub-clones will be provided on request. HDAdVs were propagated using 293FLPe cells with the addition of the FL helper virus, as previously described^36^.

### Cell culture

The hiPSC lines 246H1 (a gift from Dr Yamanaka at Kyoto University, Kyoto, Japan) and TIG3/KOSM #7 were maintained as previously described^37^. Undifferentiated hiPSCs were maintained on a feeder layer of mitomycin-treated SNL76/7 cells (DS Pharma Biomedical, Osaka, Japan) in DMEM/F12 (Nacalai Tesque, Kyoto, Japan) supplemented with 20% Knockout-Serum Replacement (Invitrogen, Carlsbad, CA, USA), 0.1 mM nonessential amino acids (Wako, Osaka, Japan), 0.1 mM β-mercaptoethanol, 50 U/mL penicillin and 50 mg/mL streptomycin (Nacalai Tesque), and 5 ng/mL bFGF (PeproTech, Rocky Hill, NJ, USA).

### Generation of the gene-targeted hiPSCs

Clumps of hiPSCs were infected with the INS-Venus HDAdV. Then, the hiPSCs were selected by 50 μg/mL G418 (Nacalai Tesque) 1 day after infection. After 3 weeks, surviving colonies were transferred to 96-well plates and GANC selection (2 μmol/L; Invitrogen) was started. After double selection, the surviving colonies were expanded and used to identify gene-targeted clones by PCR and Southern blotting analyses. To confirm genome-targeting events, DNA was digested with *Bst*Xl and analysed with 3 probes (as indicated in Fig. 1A).

For NGN3-mCherry targeting, we used INS-Venus-targeted hiPSC clone #9 after confirming that β cell differentiation was induced in this clone at a higher efficiency than in the other clones. Clumps of clone #9 cells were infected with NGN3-mCherry HDAdV, and they were selected by 0.5 μg/mL puromycin (InvivoGen, San Diego, CA, USA) 1 day after infection. After 1 week, the surviving colonies were selected with 2 μM GANC as described above and then subjected to PCR and Southern blotting analyses. For the Southern blotting analyses, we digested the genome with *Acc*I and *Age*I (Fig. 1C, [Ac, Ag]) and analysed the digested DNA with 3 probes (as indicated in Fig. 1C). The dual KI hiPSCs (*INS*-Venus/*NGN3*-mCherry) (hIveNry) will be provided upon request in accordance with the material transfer agreement (MTA).

### Pancreatic β cell differentiation from hiPSCs

*In vitro* pancreatic β cell differentiation was performed as previously reported^13^ with some modifications for the chemical screening assays. Briefly, to detect the effect of each chemical in the screening, we omitted the chemicals (10 μM forskolin, 5 μM Alk5i, 10 μM dexamethasone, 10 mM nicotinamide) from the final stages of the original protocol. The hiPSCs were plated in a V-bottom plate (Thermo Fisher Scientific, Waltham, MA, USA) at 4500 cells/well in hiPSC medium without bFGF for 3 days to form embryoid bodies and then induced for 1 day in 100 ng/mL activin A (R&D Systems, Minneapolis, MN, USA) and 3 μM CHIR (Axon Medchem, Groningen, the Netherlands). Next, the cells were induced with 100 ng/mL activin A for 2 days, then subjected to PP induction for 7 days with 1 μM dorsomorphin (Wako), 2 μM RA (Sigma-Aldrich), and 10 μM SB431542 (Cayman, Ann Arbor, MI, USA). For endocrine differentiation during the third stage of differentiation, we added the individual LOPAC chemicals (Sigma-Aldrich) at 5 μM or DMSO for control, or the cells were fully induced with 10 μM Fsk (Nacalai Tesque), 5 μM Alk5 inhibitor II (Santa Cruz, Dallas, TX, USA), 10 μM Dex (Sigma-Aldrich), and 10 mM Nico (Sigma-Aldrich) as a positive control.

### Knockdown using a shRNA system

shRNA targeting *hPDX1* and *hFGFR1* were prepared by inserting the following oligonucleotides into pLKO.1-puro and pLKO.1-DsRed, respectively: 5′ CCGG**TGACCG AGAGACACAT CAA**CTCGAG**T TGATGTGTCT CTCGGTCA**TT TTTG-3′ (into the target sequence reported as sihuPDX1 in a previous report^38^) and 5′ CCGGGATGGC ACCCGAGGCA TTATTCTCGA GAATAATGCC TCGGGTGCCA TCTTTTTG-3′ (commercially available from Sigma-Aldrich as a validated sequence). The viruses were prepared as previously reported^39^.

The hIveNry cells were infected before differentiation for 1 h in suspension and with shaking. They were then added to the medium and incubated for 1 day, before the medium was changed to human iPS medium. One or two days after, the infected hiPS cells were differentiated to PPs according to the current protocol. At day 10 or 11 after the differentiation, 1000 DsRed-positive cells were sorted into 10 μL FCP lysate (Qiagen) or total RNA was extracted from whole cells using a SV 96 kit (Promega) and subjected to quantitative real-time PCR as described below.

### Immunostaining

The hiPSCs and differentiated cells were fixed with 4% paraformaldehyde. Expression of the differentiation markers was assessed using the following antibodies: goat anti-SOX17 (R&D Systems), guinea pig anti-INS (DAKO, Glostrup, Denmark, A0564, 1:400; or Genetex, Irvine, CA, USA, 1:100), rabbit anti-GCG (DAKO, 1:100), mouse anti-GCG (Sigma-Aldrich, 1:500) goat anti-SST (Santa Cruz; 1:500), mouse anti-C-peptide (Cell Signalling, Danvers, MA, USA, 1:1000), rabbit anti-GFP (MBL, Nagoya, Japan, 1:500), mouse anti-RFP (MBL, 1:1000), and sheep anti-NGN3 (R&D Systems, 1:200), rabbit anti-CHGB (Sigma-Aldrich, 1:200). The following secondary antibodies were used: Alexa 488-conjugated goat anti-guinea pig IgG (Molecular Probes), CF488-conjugated donkey anti-guinea pig IgG (Biotium, Fermont, CA, USA, 1:500), Alexa 546-conjugated goat anti-mouse IgG (Molecular Probes), Alexa 555-conjugated donkey anti-sheep IgG (Abcam, Cambridge, UK, ab150178, 1:500), Alexa 568-conjugated donkey anti-goat IgG (Molecular Probes, 1:300), and Alexa 546-conjugated anti-rabbit IgG (Molecular Probes, 1:200). Cy5-conjugated anti-rabbit IgG, Cy3-conjugated anti-rabbit IgG, and Cy3-conjugated anti-mouse IgG were purchased from Jackson ImmunoResearch Laboratories (West Grove, PA, USA). The cells were co-stained with DAPI (Sigma-Aldrich). The samples were visualized using Leica TCS SP8 confocal microscope, Zeiss Axiovert 200 M microscope (Zeiss) and Keyence BZ-X700 fluorescence microscope (Keyence).

### Quantitative real-time PCR

RNA was extracted using an SV RNA kit and SV96 kit (Promega, Madison, WI, USA). For cDNA synthesis, we used ReverTra Ace qPCR RT Master Mix with gDNA Remover (TOYOBO, Osaka, Japan) and analysed the results with Gene Ace SYBR qPCR Mix (Nippon Gene, Tokyo, Japan) on a Light Cycler 480 thermal cycler (Roche, Basel, Switzerland). The expression level of each gene was normalised to that of GAPDH using the delta-delta CT method and expressed as arbitrary units. The primers used are listed in Supplementary Table S1. For the sorted samples, we also used a FastLane Cell cDNA kit (Qiagen, Dusseldorf, Germany). We sorted 50 target cells in each tube containing 5 μL FCP lysis buffer and, after following the manufacturer’s protocol to remove genomic DNA, performed the reverse transcription procedure. Real-time PCR was performed using the Gene Ace SYBR qPCR mix.

### C-peptide release assay

Differentiated hiPSCs were washed 3 times with phosphate-buffered saline (PBS) and pre-incubated with KRBH containing 2.8 mM glucose for 1 h. The cells were washed 3 times with PBS and incubated with low glucose (2.8 mM)/KRBH buffer for 1 h. Finally, the cells were incubated with high glucose (25 mM)/KRBH buffer for 1 h. INS secretion into the culture medium was measured with a Human Ultrasensitive C-peptide ELISA kit (Mercodia, Uppsala, Sweden).

### High-end imaging analysis

In the screening assay, differentiated cells in a V-bottom plate were stained with Hoechst 33342 (Molecular Probes). This signal was imaged, together with Venus and mCherry fluorescence via BGRFR_386, BGRFR_485 and BGRFR_549 filters, respectively, using a 5x objective and the same exposure time in all plates in an ArrayScan system (Thermo Fisher Scientific) running HCS scan software and the CellHealth profiling algorithm protocol.

### Flow cytometry

The differentiated cells were dissociated with Accutase (Nacalai Tesque). The number of Venus- and mCherry-expressing cells was analysed in an SH800Z cell sorter (Sony, Tokyo, Japan). The Venus+ cells, mCherry+ cells, and negative cells were sorted for mRNA expression analysis by excluding dead cells with 7AAD and doublet cells with a FSC-H/FSC-W chart.

### Statistical analysis

Statistical analysis was performed using paired t-tests. Differences between groups were considered significant for values of p < 0.05.

## Additional Information

**How to cite this article**: Yamashita-Sugahara, Y. *et al.* An inhibitor of fibroblast growth factor receptor-1 (FGFR1) promotes late-stage terminal differentiation from NGN3+ pancreatic endocrine progenitors. *Sci. Rep.* **6**, 35908; doi: 10.1038/srep35908 (2016).

**Publisher’s note:** Springer Nature remains neutral with regard to jurisdictional claims in published maps and institutional affiliations.

## Supplementary Material

Supplementary Video

Supplementary Information
