# Supplementary material for: An inhibitor of fibroblast growth factor receptor-1 (FGFR1) promotes late-stage terminal differentiation from NGN3+ pancreatic endocrine progenitors
Source: Sci Rep. 2016 Oct 27;6:35908. doi: 10.1038/srep35908 (PMC5081516; doi:10.1038/srep35908)
Supplement: Supplementary Information [file srep35908-s2.doc]

**An inhibitor of fibroblast growth factor receptor-1 (FGFR1) promotes late-stage terminal differentiation from NGN3+ pancreatic endocrine progenitors**

Yzumi Yamashita-Sugahara^1^, Masahito Matsumoto^1^, Manami Ohtaka^2^, Ken Nishimura^3^, Mahito Nakanishi^2^, Kohnosuke Mitani^4^, Yasushi Okazaki^1*^

^1^Division of Functional Genomics and Systems Medicine, Research Center for Genomic Medicine, Saitama Medical University, Saitama, Japan

^2^Biotechnology Research Institute for Drug Discovery

National Institute of Advanced Industrial Science and Technology (AIST), Ibaraki, Japan

^3^Laboratory of Gene Regulation, Faculty of Medicine, University of Tsukuba

Ibaraki, Japan

^4^Division of Gene Therapy, Research Center for Genomic Medicine, Saitama Medical University, Saitama, Japan

*Correspondence and request for materials should be addressed to

M.D., Ph.D., Yasushi Okazaki

Division of Functional Genomics and Systems Medicine,

Division of Translational Research,

Research Center for Genomic Medicine,

Saitama Medical University

1397-1 Yamane, Hidaka, Saitama 350-1241, Japan

E-mail: [okazaki@saitama-med.ac.jp](mailto:okazaki@saitama-med.ac.jp)

Tel.: +81 42 984 0318

Fax: +81 42 984 0349


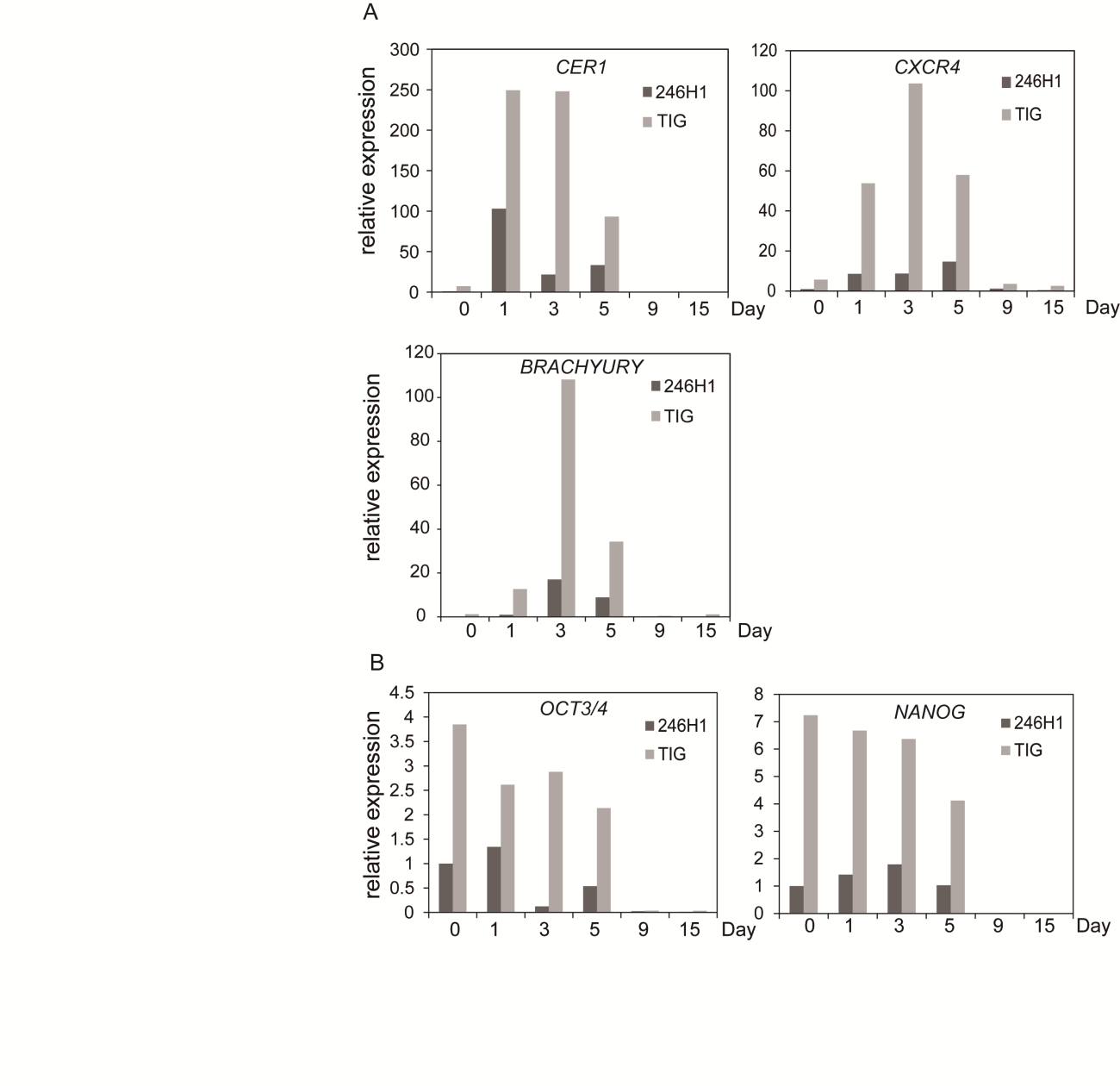


**Figure S1. Comparison of pancreatic endocrine differentiation between the hiPSC lines 246H1 and TIG3KOSM7 (TIG).**

246H1 and TIG cells were differentiated for 15 days according to previous methods[^10^](#_ENREF_10). (A) The mRNA expression levels of markers of mesendoderm (*BRACHYURY*), definitive endoderm (*CER1* and *CXCR4*), and (B) pluripotency (*OCT3/4*[*POU5F1*] and *NANOG*) were analysed by quantitative RT-PCR. *CER1*: Cerberus 1, DAN family BMP antagonist; *CXCR4*: chemokine (C-X-C motif) receptor 4; *OCT3/4* (also known as *POU5F1*): POU class 5 homeobox 1; *NANOG*: Nanog homeobox.


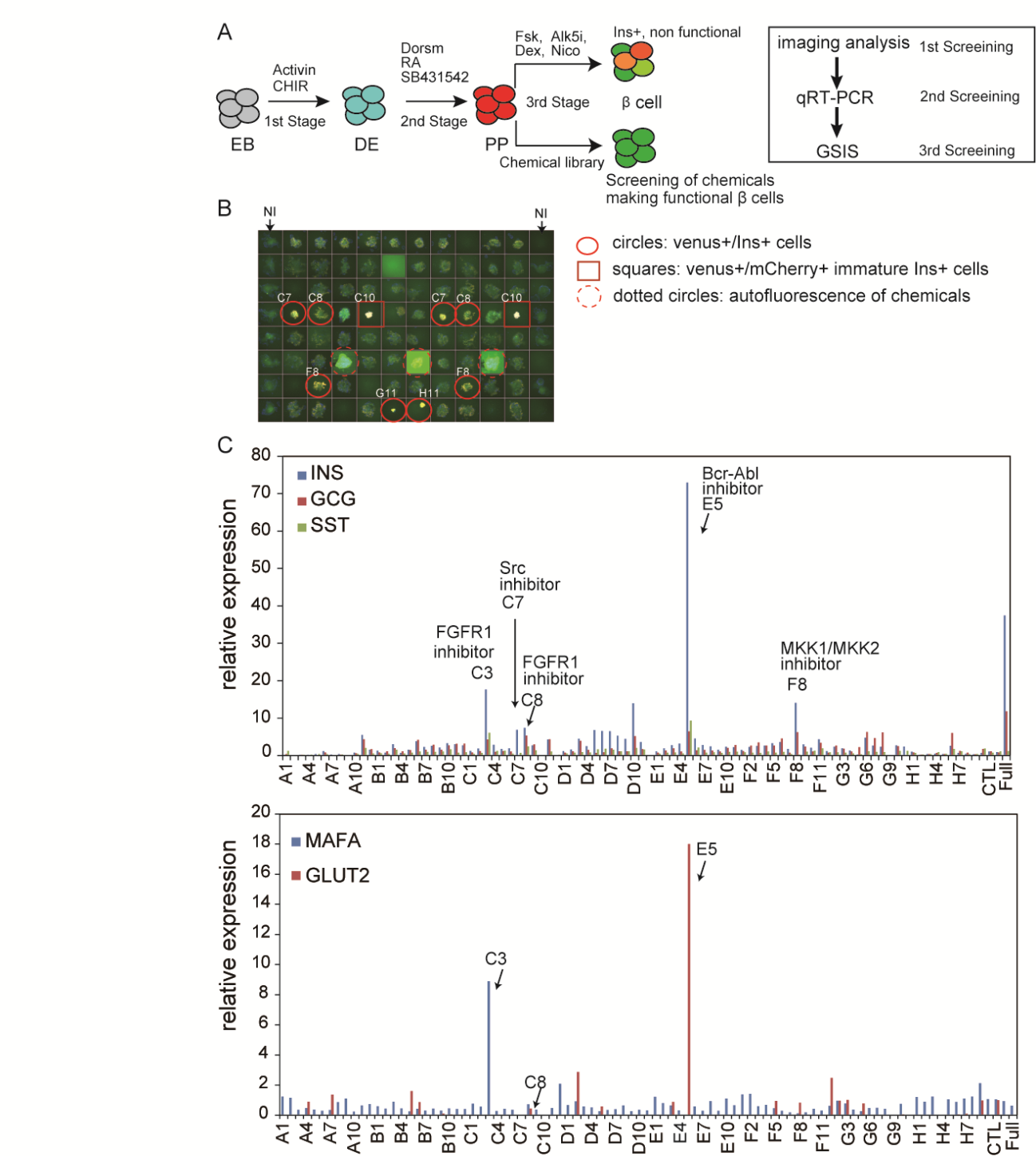


**Figure S2. Screening of 90 chemicals during pancreatic β cell differentiation.**

(A) The differentiation methods for screening are illustrated. At the third stage, we added each chemical for screening, without the 4 chemicals of the original protocol, or solvent-containing medium as a control or the cells were fully induced with the 4 chemicals as a positive control. Dorsm: dorsomorphin; RA: retinoic acid; Fsk: forskolin; Alk5i: Alk 5 inhibitor II; Dex: dexamethasone; Nico: nicotinamide. The workflow for chemical screening was as follows: (B) on day 21 (final day of the third stage), the differentiated β cells were analysed with a high-end imaging analyser *in vivo*, (C) and total RNA was extracted from 2 to 4 clusters to analyse the mRNA levels of the β cell markers. Finally, the C-peptide release assay (GSIS) was performed with the selected candidate chemicals. (B) This image indicates the first step of screening: the circles indicate positively promoted differentiation with Venus+/Ins+ cells; the squares indicate immature Ins+ cells; some of the chemicals were auto-fluorescent (dotted circles). There was no differentiated negative control related to NI. The data are from 1 of the 3 independent assays.


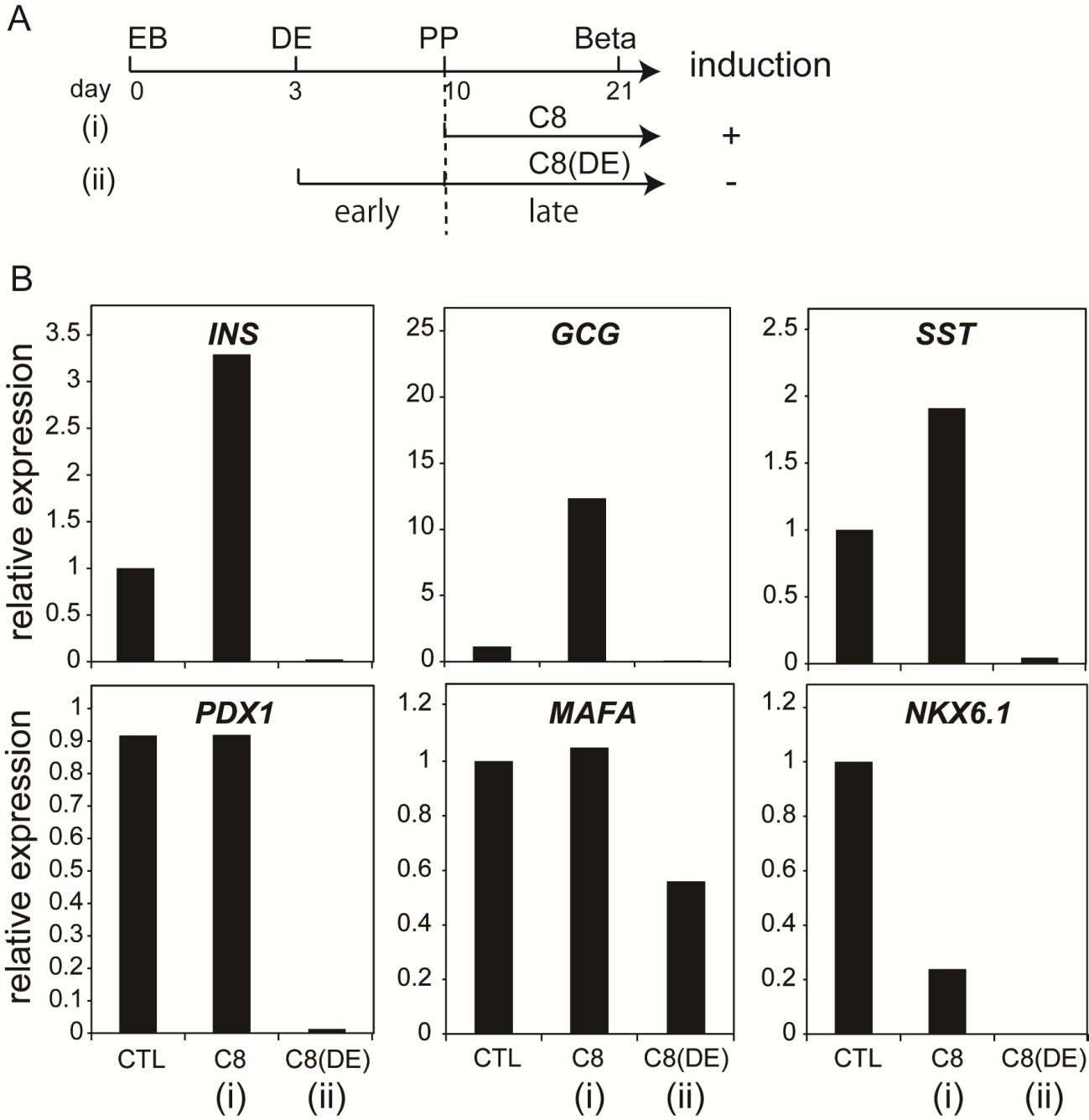


**Figure S3. Treatment with C8 in an early stage inhibits β cell differentiation.**

(A) A schematic illustration of the stage of β cell differentiation showing C8 treatment at an early (DE) (ii) or late (intermediate-terminal) stage of differentiation (i). Successful differentiation is indicated as a plus (+) and failed differentiation is indicated as a minus (-). (B) The mRNA expression levels of the endocrine markers in the 2 conditions analysed ([i], [ii]). The experiment was performed twice, and the result represents the average. n = 2, the other experiment was performed at another time and analysed by FACS analysis and showed similar results (data not shown).


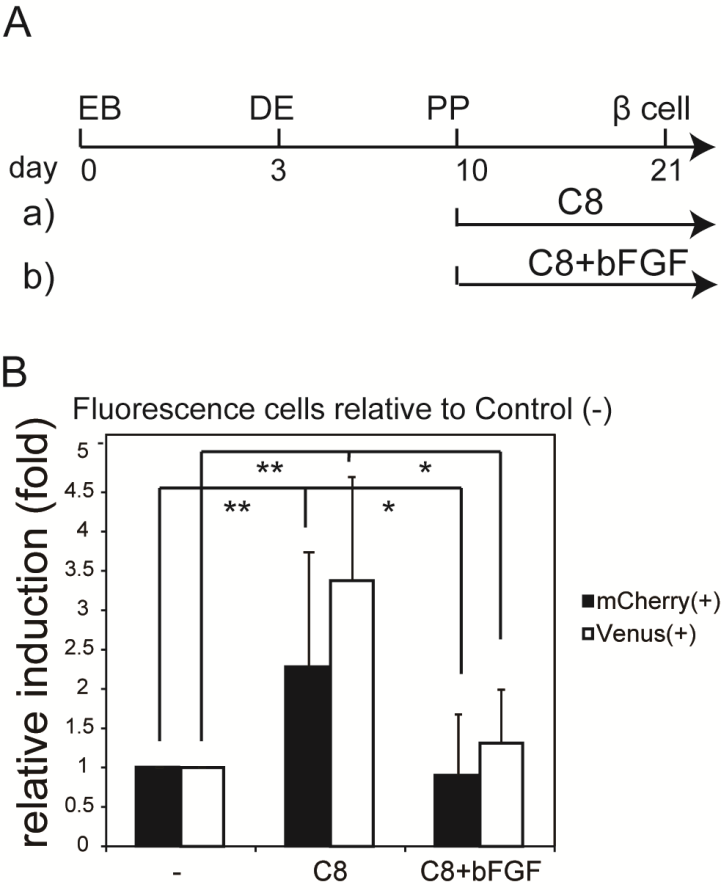


**Figure S4. C8 treatment promotes increased mCherry+ EP and Venus+ β cell populations.**

(A) A schematic of the β cell differentiation procedure is shown, in which C8 or C8 + bFGF were added at the late stage (10–21 days). (B) The graph indicates the percentage of the Venus or mCherry population on day 21 of differentiation, which was calculated relative to the control. Error bars indicate standard deviation, n = 3. *p < 0.05; **p < 0.01; ***p < 0.001.


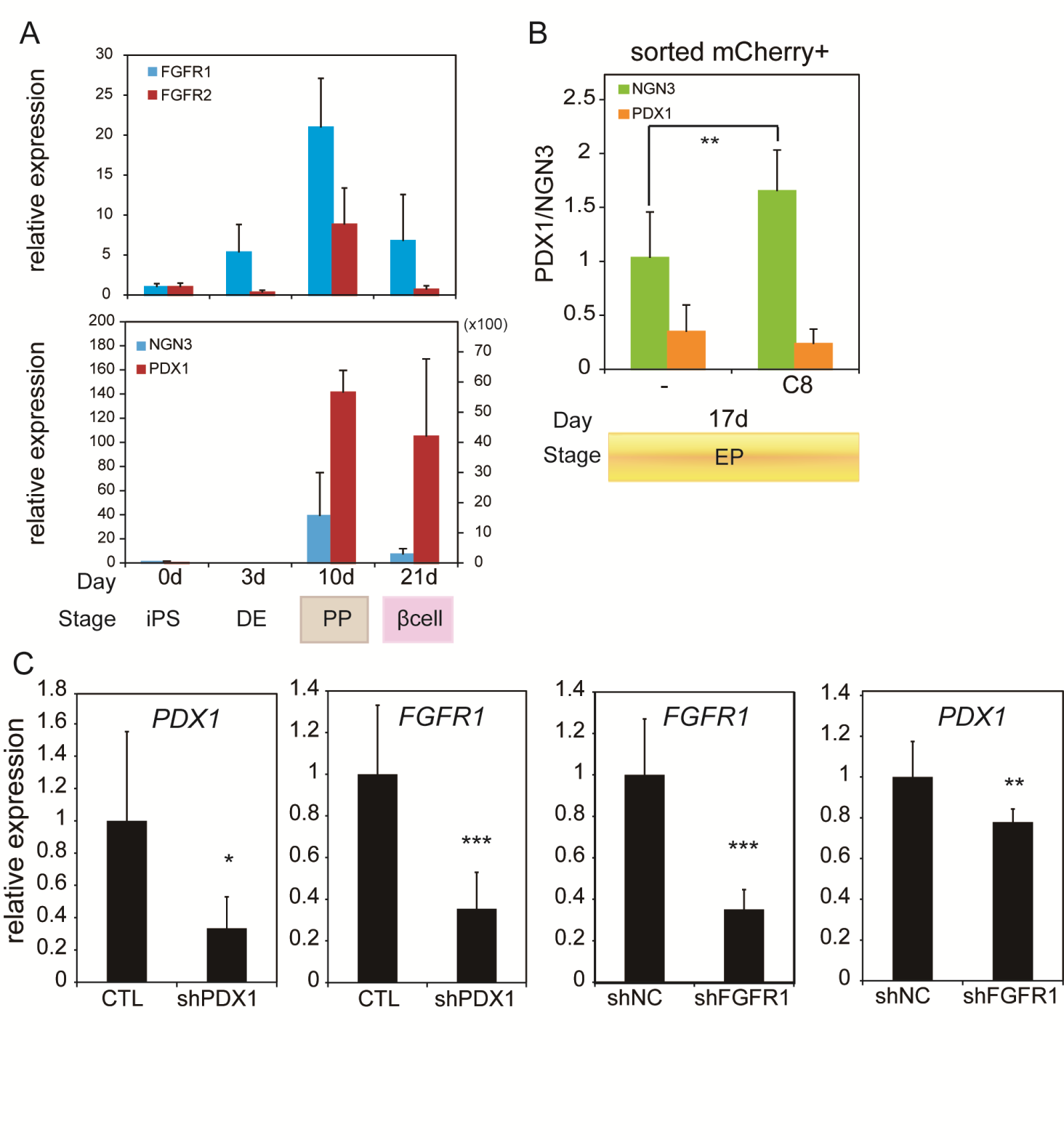


**Figure S5. *PDX1* and *FGFR1* expression is correlated during β cell differentiation and the reciprocal expression of *PDX1* and *NGN3* at the EP stage.**

(A) mRNA expression analysis of *FGFR1*, *FGFR2*, *PDX1*, and *NGN3* during β cell differentiation at the indicated days. (B) On day 17 of differentiation, NGN3+ EP mCherry+ cells were sorted, and the mRNA expression levels *of NGN3* and *PDX1* were analysed; the data indicate the relative expression of *PDX1* compared to *NGN3*. (C) The knockdown assay was performed at the early stage of differentiation using lentivirus expressing *shPDX1* or *shFGFR1*. The hiPSCs (hIveNry) were infected with lentivirus prior to the induction of differentiation and the cells were subjected to cell sorting (for *shPDX1*) at day 10 or 11. For *shFGFR1*, total RNA was extracted without sorting and the *PDX1* and *FGFR1* mRNA expression levels were analysed.

**
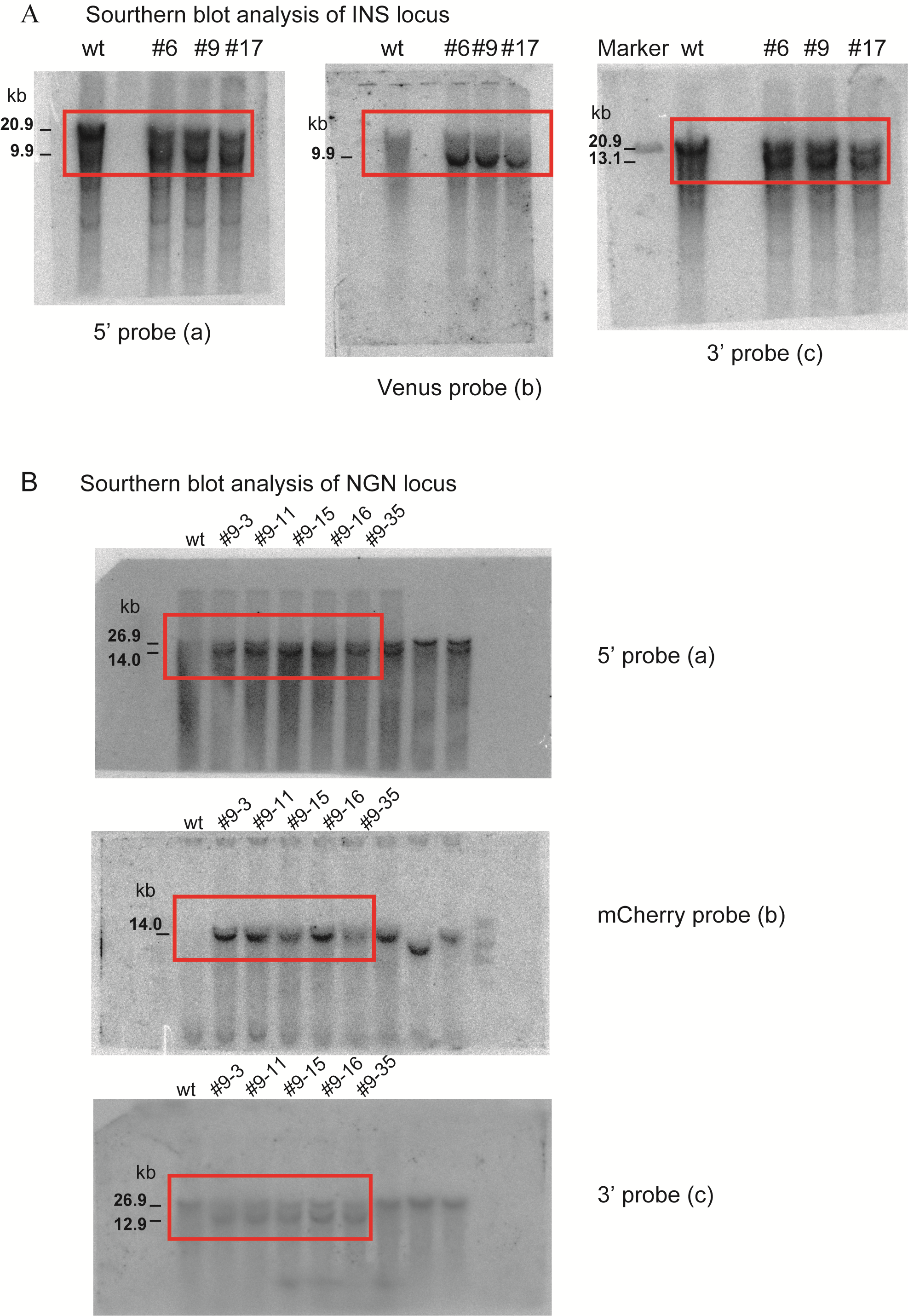
**

**Figure S6.** **Uncropped image of Southern Blot analysis shown in Figure 1:**

(A)Full-length blots used in Figure 1B showing *INS* locus hybridized by 5’-, Venus, and 3’- probes, and (B) Full-length blots used in Figure 1D showing *NGN* locus hybridized by 5’-, mCherry and 3’- probes.

**Supplementary video. Live imaging of hIveNry cells during β cell differentiation from 19 to 21 days.**

The hIveNry cells were differentiated into β cells. The live imaging analysis was performed by Leica SP8 confocal microscope (Leica) from 19 to 21 days of differentiation by capturing once in every 30 minute.

**Supplementary Table S1.**

**The primers** used for mRNA expression analysis

| HsCER1_537F | TCCTGCTTCCCATGAGACTT |
| --- | --- |
| HsCER1_628R | AATGAACAGACCCGCATTTC |
| HsCXCR4_91F | TTACCATGGAGGGGATCAGT |
| HsCXCR4_178R | CAGGGTTCCTTCATGGAGTC |
| HsGAPDH_F | ATGTTCGTCATGGGTGTGAA |
| HsGAPDH_R | TGTGGTCATGAGTCCTTCCA |
| HsPOU5F1_L631 | CTTTGAGGCTCTGCAG |
| HsPOU5F1_R744 | TCTGCTTTGCATATCT |
| HsBrachyury_R1422 | CATGCAGGTGAGTTGT |
| HsBrachyury_RL1317 | GCTGTGACAGGTACCC |
| HsNANOG_L599 | TCTCCAACATCCTGAACCTCA |
| HsNANOG_R704 | TTGCTATTCTTCGGCCAGTT |
| HsPDX1_460F | CCTTTCCCATGGATGAAGTC |
| HsPDX1_555R | CGTCCGCTTGTTCTCCTC |
| HsINS_31F | GCCATCAAGCAGATCACTGT |
| HsINS_149R | CAGGTGTTGGTTCACAAAGG |
| HsGCG_264F | GCATTTACTTTGTGGCTGGA |
| HsGCG_368R | CCTGGGAAGCTGAGAATGAT |
| HsSST_206F | CCCCAGACTCCGTCAGTTTC |
| HsSST_313R | TCCGTCTGGTTGGGTTCAG |
| HsGLP1R_F | GAGGTCATCTTTGCCTTTGTG |
| HsGLP1R_R | TAATATGGCCACCATCAGCC |
| HsNKX6.1_F | ATTCGTTGGGGATGACAGAG |
| HsNKX6.1_F | TGTCTCCGAGTCCTGCTTCT |
| HsSLC2A2_F | TGCATTCAGCAATTGGACC |
| HsSLC2A2_R | AGCACTCCAGCAAAGAGGAA |
| HsNGN3_205Fw | TCGCTGCTCATCGCTCTCTAT |
| HsNGN3_294Rv | CTCACGGGTCACTTGGACAGT |
| mCherryF148 | AAGCTGAAGGTGACCAAGGG |
| mCherryR264 | CAAGTAGTCGGGGATGTCGG |
| GFP 3F539 | CCGACCACTACCAGCAGAAC |
| GFP 3R672 | GAACTCCAGCAGGACCATGT |
| HsGCK_1F | GTGTACAAGCTGCACCCCA |
| HsGCK_1R | TCCGACTCGATGAAGGTGAT |
| HsKCNK3_3F | TAGGTCTCCCACCTTCCCTT |
| HsKCNK3_3R | CTGGGTTTCCACTTTCTCCA |
| HsPTF1A_1F | CAGAAGGTCATCATCTGCCA |
| HsPTF1A_1R | CAGACTTTGGCTGTTCGGAT |
| HsSLC30A8_1F | GGCCGTCATGGAGTTTCTT |
| HsSLC30A8_1R | CACCGGTTTCTGTTGGAGTT |
| HsUCN3_1F | GAGGGAAGTCCACTCTCGG |
| HsUCN3_1R | TGTAGAACTTGTGGGGGAGG |
| HsABCC8_739_F | CTGCTGTCCAAAGGCACCTA |
| HsABCC8_906_R | CTGAATGTCCTTCCGCACCT |

**Supplementary Table S2**

**List of LOPAC chemicals**

| ROW | COLUMN | ITEM | COMPOUND | SELECTIVITY |
| --- | --- | --- | --- | --- |
| A | 1 | PZ0001 | Atorvastatin calcium salt trihydrate | HMG-CoA reductase |
| B | 1 | PZ0014 | Linezolid |  |
| C | 1 | PZ0107 | CP-101537 | MMP |
| D | 1 | PZ0121 | CP-135807 | 5-HT1D |
| E | 1 | PZ0138 | SC-51089 hydrate | EP1 |
| F | 1 | PZ0151 | PF-956980 | FGFR1 |
| G | 1 | PZ0174 | Sonepiprazole | D4 |
| H | 1 | PZ0187 | GSI-953 | ß-secretase |
| A | 2 | PZ0002 | Maraviroc | CCR-5 |
| B | 2 | PZ0015 | Trovafloxacin |  |
| C | 2 | PZ0108 | CP-335963 | Aurora-2 |
| D | 2 | PZ0123 | Avridine |  |
| E | 2 | PZ0139 | SC-58125 | COX2 |
| F | 2 | PZ0152 | UK-356618 | MMP3 |
| G | 2 | PZ0175 | PD-149163 | NTR1 |
| H | 2 | PZ0189 | CP-316819 | glycogen phosphorylase |
| A | 3 | PZ0003 | Sildenafil | PDE5 |
| B | 3 | PZ0016 | Dofetilide | Ikr |
| C | 3 | PZ0109 | PD-161570 | FGFR-1 |
| D | 3 | PZ0124 | CP-66713 | ADRA2 |
| E | 3 | PZ0140 | EBPC | Aldose Reductase |
| F | 3 | PZ0155 | PF-03716556 | H,K-ATPase |
| G | 3 | PZ0176 | SC-26196 | Delta6D |
| H | 3 | PZ0190 | Avasimibe | Acyl-CoA:Cholesterol O-Acyltransferase |
| A | 4 | PZ0004 | Varenicline | nAChRa7 |
| B | 4 | PZ0020 | Temsirolimus | mTOR |
| C | 4 | PZ0110 | SC-57461A | LTA4 |
| D | 4 | PZ0125 | CP-53631 | Serotonin |
| E | 4 | PZ0141 | PD-156707 | Endothelin Receptor |
| F | 4 | PZ0156 | UK-383367 | PCP |
| G | 4 | PZ0177 | OSU6162 | Dopamine |
| H | 4 | PZ0191 | Crizotinib | c-MET / ALK |
| A | 5 | PZ0005 | Voriconazole |  |
| B | 5 | PZ0021 | Tigecycline |  |
| C | 5 | PZ0111 | PD-407824 | Wee1/Chk |
| D | 5 | PZ0127 | CP-74416 | DNA Gyrase |
| E | 5 | PZ0142 | PD-180970 | Bcr-Abl |
| F | 5 | PZ0158 | PF-3845 | FAAH |
| G | 5 | PZ0178 | PHA 767491 | cdc7/cdk9 |
| H | 5 | PZ0192 | Bosutinib | Src/Abl dual inhibitor |
| A | 6 | PZ0006 | Exemestane | Aromatase |
| B | 6 | PZ0022 | BLI-489 | b-lactamase |
| C | 6 | PZ0112 | PD-184161 | MEK |
| D | 6 | PZ0129 | CP-380736 | EGFR |
| E | 6 | PZ0143 | PF-4708671 | S6K1 |
| F | 6 | PZ0159 | MCOPPB | ORL1 |
| G | 6 | PZ0179 | Valdecoxib | COX-2 |
| H | 6 | PZ0193 | Axitinib | VEGFR 1, 2, 3 |
| A | 7 | PZ0007 | Azithromycin |  |
| B | 7 | PZ0100 | CP-154526 | CRF1 |
| C | 7 | PZ0113 | PD173952 | Src |
| D | 7 | PZ0130 | CP-64434 | HDAC |
| E | 7 | PZ0144 | PD-161989 | AMPA |
| F | 7 | PZ0160 | UK-5099 |  |
| G | 7 | PZ0180 | Delavirdine | Reverse transcriptase |
| H | 7 | PZ0118 | Nafoxidine | Estrogen Receptor |
| A | 8 | PZ0008 | Celecoxib | COX-2 |
| B | 8 | PZ0101 | CP-226269 | D4 |
| C | 8 | PZ0114 | PD-166866 | FGFR1 |
| D | 8 | PZ0131 | PF-998425 | Androgen receptor |
| E | 8 | PZ0146 | SC-53116 | 5-HT4 |
| F | 8 | PZ0162 | PD 0325901 | MKK1 / MKK2 |
| G | 8 | PZ0181 | PD184352 | MEK |
| H | 8 | PZ0137 | CP-471474 | MMP |
| A | 9 | PZ0009 | Tolterodine | CHRM3 |
| B | 9 | PZ0102 | CP-93129 | 5-HT1B |
| C | 9 | PZ0115 | CP-31398 | p53 |
| D | 9 | PZ0132 | SC-51322 | EP2 |
| E | 9 | PZ0147 | PHA-665752 | c-MET / ALK |
| F | 9 | PZ0170 | Torcetrapib | CTEP |
| G | 9 | PZ0182 | Pactamycin |  |
| H | 9 | PZ0150 | SU5614 | FLT3 |
| A | 10 | PZ0011 | Eletriptan | 5-HT1B/1D |
| B | 10 | PZ0103 | CP-346086 | MTP, MTTP |
| C | 10 | PZ0116 | PD-166285 | Src, FGFR |
| D | 10 | PZ0135 | PHA-543613 | nAChRa7 |
| E | 10 | PZ0148 | CP-802079 | GlyT1 |
| F | 10 | PZ0171 | CP-100356 | MDR1 |
| G | 10 | PZ0183 | ERB-041 | Estrogen Receptor |
| H | 10 | PZ0173 | CP-775146 | PPARa |
| A | 11 | PZ0012 | Sunitinib | RTKs |
| B | 11 | PZ0104 | CP-91149 | glycogen phosphorylase |
| C | 11 | PZ0117 | PF-573228 | FAK |
| D | 11 | PZ0136 | CP-100263 | NK-1 |
| E | 11 | PZ0149 | CP-868388 | PPARa |
| F | 11 | PZ0172 | Gisadenafil | PDE5 |
| G | 11 | PZ0185 | PF-431396 | PYK2/FAK |
| H | 11 | PZ0186 | PF-477736 | Chk1 |
| A | 12 | PZ0013 | Nelfinavir | Protease |
| B | 12 | PZ0106 | SC-236 | COX-2 |
